# Towards Fingerprint Mosaicking Artifact Detection: A Self-Supervised Deep Learning Approach

**DOI:** 10.3390/s26123684

**Published:** 2026-06-09

**Authors:** Laurenz Ruzicka, Alexander Spenke, Stephan Bergmann, Gerd Nolden, Bernhard Kohn, Clemens Heitzinger

**Affiliations:** 1 Department of Digital Safety and Security, Austrian Institute of Technology, 1210 Vienna, Austria; bernhard.kohn@ait.ac.at; 2 Federal Office for Information Security, Bundesamt für Sicherheit in der Informationstechnik, 53175 Bonn, Germany; alexander.spenke@bsi.bund.de (A.S.); stephan.bergmann@bsi.bund.de (S.B.); gerd.nolden@bsi.bund.de (G.N.); 3 Institute of Mathematics, Faculty II—Mathematics and Natural Sciences, Technische Universität Berlin, 10623 Berlin, Germany; 4 Weierstraß Institute for Applied Analysis and Stochastics (WIAS), 10117 Berlin, Germany; heitzinger@wias-berlin.de

**Keywords:** contactless fingerprint, mosaicking artifacts, detection

## Abstract

Fingerprint mosaicking—the process of combining multiple fingerprint impressions into a single master fingerprint—is an essential step in modern biometric systems, but it is prone to errors that can significantly degrade image quality. This paper proposes a deep learning-based approach to detect and score hard mosaicking artifacts in fingerprint images. Our method uses a self-supervised learning framework to train a segmentation model on large-scale unlabeled fingerprint data, eliminating the need for manual artifact annotation. The proposed model effectively identifies mosaicking errors, achieving high segmentation performance across multiple fingerprint modalities—contactless, rolled, and pressed—and proves robust to different data sources. We also introduce a mosaicking artifact score that quantifies the severity of detected errors and enables automated evaluation of fingerprint images at scale. Training and evaluation rely on synthetic artifacts, we therefore provide a qualitative comparison to real stitching failures and discuss the limits of this validation strategy in detail. By addressing the previously underexplored problem of reference-free hard-artifact detection in fingerprints, our work contributes to improving the accuracy and reliability of fingerprint-based biometric systems.

## 1. Introduction & Related Work

Fingerprints have long been established as a critical biometric trait for personal identification and verification, because of their uniqueness and permanence [1]. They are used in a wide range of applications, from law enforcement and border control to unlocking personal devices and securing sensitive information. The accuracy of such systems depends heavily on the quality of the captured fingerprint images, which are typically acquired using either contact-based [2] or contactless [3] methods. To capture full rolled fingerprints, multiple partial impressions are combined through mosaicking [4,5,6], a process also used in domains such as photography and medical imaging [7,8,9].

Errors in mosaicking can distort minutiae, potentially causing misidentification [10]. We categorize these errors as soft, describing local deformations that preserve ridge continuity, and hard, denoting clear misalignments in the ridge valley structure that are sometimes partially concealed by blending. Detecting hard errors is crucial, yet current fingerprint systems lack tools to identify them reliably.

Fingerprint mosaicking has been studied extensively. Early work by Jain and Ross [11] and Choi et al. [12] emphasized accurate alignment of impressions. Later methods explored both feature- and image-based approaches [13], contactless acquisition [10], and deep learning-based mosaicking [4]. Minutia-free techniques have also been proposed [6].

Related work on detecting mosaicking artifacts exists in video stitching [14], panoramic quality evaluation [15], and view synthesis assessment [16], often using metrics such as local sharpness [17]. When reference images are available, methods such as structural similarity [18], geometric accuracy [19], and spectral validation [20] are applicable.

Despite these advances, no method currently targets reference-free detection of hard mosaicking errors in fingerprints. We address this gap with a deep learning-based detector that uses a self-supervised pipeline for training and evaluation across diverse modalities. We additionally propose a new artifact score and assess the effect of mosaicking errors on identification and authentication performance.

### Contribution

We add to the existing work by proposing a framework for detecting hard fingerprint mosaicking artifacts. Our contributions are:

- A deep learning model for hard stitching artifact detection, together with a self-supervised data annotation pipeline and extensive data augmentation.
- Release of the deep learning framework’s code, including the annotation pipeline, under the Mozilla Public License Version 2.0.
- Evaluation of model performance on out-of-distribution sensor modalities.
- An analysis of model robustness across different training modalities and dataset sizes, and robustness on synthetic fingerprints with quality alterations simulating wounds, scars, and similar distortions.
- A mosaicking artifact score with an explicit physical interpretation and parameter justification.
- A qualitative comparison between synthetic training artifacts and real mosaicking failures observed in the ROD-1 dataset, with an honest assessment of the limits of synthetic-only validation.
- A controlled baseline comparison against five segmentation configurations (plain UNet, MAnet, Linknet, and FPN decoders on the same encoder, plus an encoder swap to ResNet-50) under matched training conditions.
- An assessment of the impact of mosaicking errors on identification and authentication performance.

## 2. Methods

### 2.1. Data

We used a real-world contactless fingerprint dataset described by Weissenfeld et al. [21], consisting of 245,193 images from 539 users for training, 30,650 for validation, and 30,649 for testing. These are single-shot, artifact-free fingerprints used as ground truth. A subset of 500 images from 50 fingers (10 images per finger) was used to evaluate the impact of artifact removal via Equal Error Rate (EER) calculations. The subset size and its statistical implications are discussed in Section 3.4.

For cross-modality testing, we used the NIST Special Publication 300a dataset [22], which includes paired rolled and slap fingerprints.

We also trained a second model with the same architecture and hyperparameters (except the learning rate) on a pressed fingerprint dataset (PRD-1) captured at the Biometric Evaluation Center (BEZ) with FTIR sensors, comprising 32,800 images from 288 identities: 80% for training, 10% for validation, and 10% for testing (PRD-1-Test) [23]. These images are assumed to be artifact-free. Additionally, rolled prints from the same subjects were collected (ROD-1). A follow-up dataset, PRD-2, includes 5495 FTIR-based pressed fingerprints from 143 subjects, 117 of whom overlap with PRD-1.

**Figure 1:**
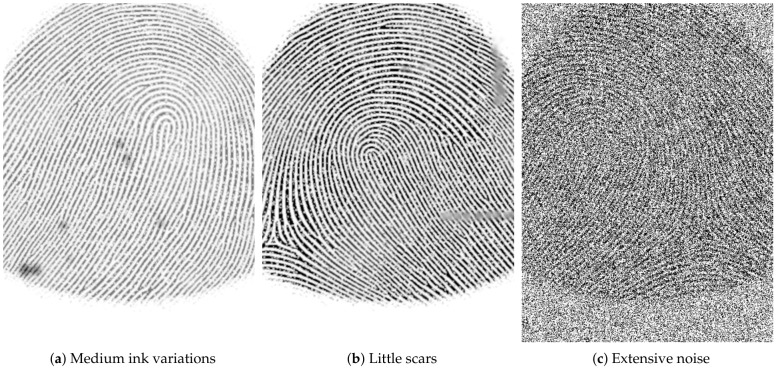
Synthetic fingerprint alterations based on SynColFinGe applied to SFinGe generated fingerprints.

To assess robustness, we used 100 synthetically generated SFinGe [24] images, modified using selected fingerprint alteration methods from [25], simulating damage, contrast changes, noise, and other distortions. A selection of those can be seen in Figure 1 and a more detailed analysis of the alternation patterns can be found in Appendix B.

### 2.2. Model

We designed a self-supervised deep learning model for detecting fingerprint mosaicking artifacts. It learns from unlabeled, artifact-free images using artificial artifact insertion to generate training signals. The code is available under the following link (https://git-service.ait.ac.at/dsai-idm/open-source/stitching-score/, accessed on 3 June 2026) [26].

#### 2.2.1. Data Pre-Processing

All images were resized to 224 × 224 pixels, balancing detail and computational load, and aligning with the pretrained backbone.

#### 2.2.2. Data Augmentation

Before artifact insertion, images were randomly augmented using:

- Random Resizing and Cropping
- Random Horizontal Flips
- Random Rotations
- Random Perspective Changes
- Gaussian Blurring
- Random Solarization
- Random Posterization
- Random Histogram Equalization

Augmentations were applied in random order to improve robustness.

#### 2.2.3. Self-Supervised Learning

**Figure 2:**
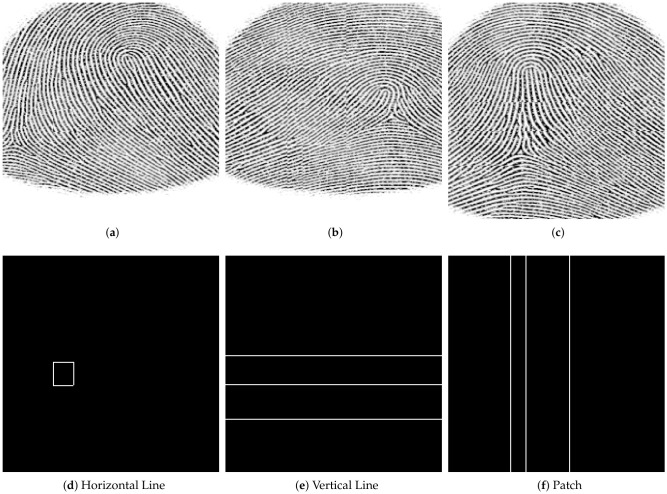
Depiction of different artifact types created for supervisory signal. Top row shows fingerprint images after adding the generated artifacts and the bottom row the corresponding artifact labels.

We applied two types of artificial artifacts during training. The first type was patch-based artifacts, where up to four rectangular patches, each covering 5–15% of the image area, were randomly placed and shifted by 2–7% of the image width or height. This can be seen in the example given in Figure 2a,b. The second type involved line-based artifacts, introduced with a probability of 25%. In this case, up to four horizontal (depicted for one example in Figure 2b,e or vertical (depicted for one example in Figure 2c,f lines of pixels—entire rows or columns—were displaced by a small offset to simulate stitching errors. These artifacts provided a diverse and realistic training signal while requiring no manual annotation.

**Figure 3:**
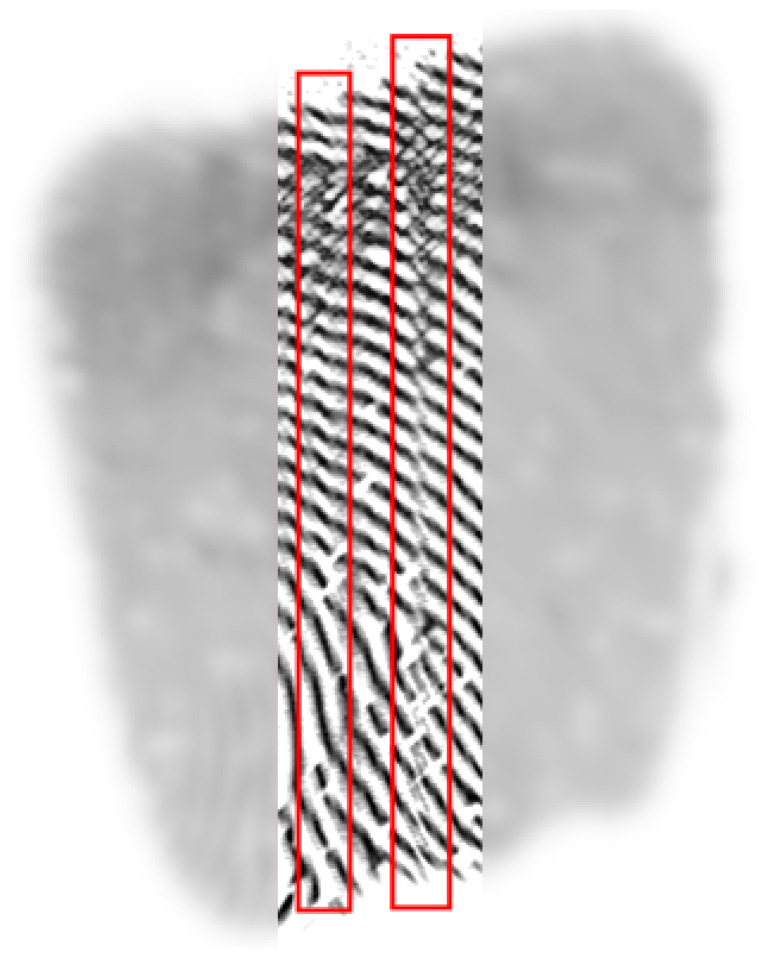
Real mosaicking failure observed on a rolled fingerprint from our acquisition setup. The two red rectangles mark thin vertical bands along which the ridge flow does not connect across the strip boundaries—a typical strip-stitch failure produced when adjacent acquisition strips are concatenated with a small horizontal misregistration. This is precisely the failure mode of our vertical-line synthetic artifact (Figure 2f) is designed to reproduce. The fingerprint ridge texture has been retained for illustration; in all downstream figures of this manuscript, real fingerprint regions are blurred or anonymized.

Figure 3 shows a real rolled fingerprint from our acquisition setup in which the stitching algorithm has produced two thin vertical bands of ridge discontinuity (red rectangles): along each band, the ridge flow on the left and right sides does not connect, even though it would join continuously in an artifact-free print. This is an example of a strip-stitch failure, in which neighboring 1D linescan strips, or partial impressions taken at adjacent rolling positions, were concatenated with a small horizontal misregistration. The visible signature in the image is exactly what our vertical-line synthetic artifact is designed to reproduce (Figure 2f). The same logic applies to the horizontal-line case (rolled-too-fast or missed-frame failures, producing row-wise discontinuities) and to the patch case (translational drift of a partial impression relative to its neighbors, producing a displaced rectangular region of ridges).

#### 2.2.4. Architecture and Hyperparameter

**Figure 4:**
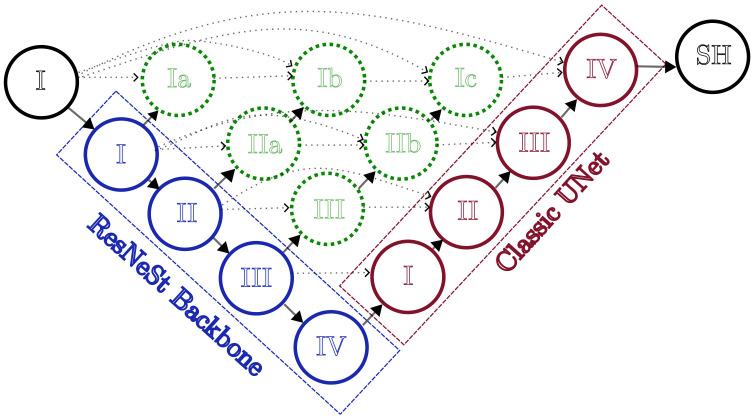
Model architecture of our proposed combination of ResNeSt for the encoder and UNet++ for the general model architecture and decoder design.

The model uses UNet++ [27] with a ResNeSt-50d encoder (via Torch Segmentation Models [28,29]), pretrained on ImageNet. Figure 4 illustrates the architecture. The encoder backbone is shown in blue on the left and the UNet++ decoder in green and red on the right. The circles represent different layers, with their colors grouping them as part of the encoder, decoder, or auxiliary layers. Dashed lines illustrate skip connections, while downward-pointing arrows indicate spatial downsampling and upward-pointing arrows signify spatial upsampling.

We deliberately adopted established, well-tested components—ResNeSt-50d as the encoder and UNet++ as the decoder—rather than proposing a new backbone. This makes the framework directly reproducible by other groups without requiring a custom architecture implementation. Also, it isolates the effect of the proposed self-supervised signal. Any performance observed can be attributed to the training scheme and the score, not to architectural novelty. The combination of split-attention features in ResNeSt-50d and the nested skip pathways of UNet++ is, however, particularly well suited to detecting small, locally coherent disturbances against a textured fingerprint background, which is the failure mode of interest here.

The model’s input is initially processed through convolution and pooling layers, represented by the black circle with an ‘I’ at the top left. It then undergoes feature extraction within the ResNeSt encoder. Intermediate outputs from the ResNeSt layers are fed into the UNet++ decoder through two mechanisms:

1. they are connected to decoder layers with matching spatial resolution, similar to the skip connections in the original UNet architecture by Ronneberger [30], and
2. they are upsampled and passed into intermediate processing layers (shown as green dashed layers), which are also linked to the decoder stack via skip connections.

After traversing the entire encoder and decoder stacks, including all intermediate layers, the processed input reaches the final segmentation head, indicated by the black circle with ’SH’ on the right. The segmentation head then produces the predicted segmentation mask as model output.

##### Learning Rate and Optimizer

We set the learning rate to $1.7\times 10^{-3}$ and employ the Stochastic Gradient Descent (SGD) optimizer with momentum. Momentum is set to 0.9 to enhance the convergence speed and stability of the training process. For the second model trained on PR recordings, we increased the learning rate to $1\times 10^{-2}$ to speed up the training process.

##### Loss Function

We use the Jaccard loss, also known as the Intersection over Union (IoU) loss, which is well-suited for segmentation tasks [31]. This loss function measures the overlap between the predicted and true segmentation masks.

##### Training Hyper-Parameters

**Figure A1:**
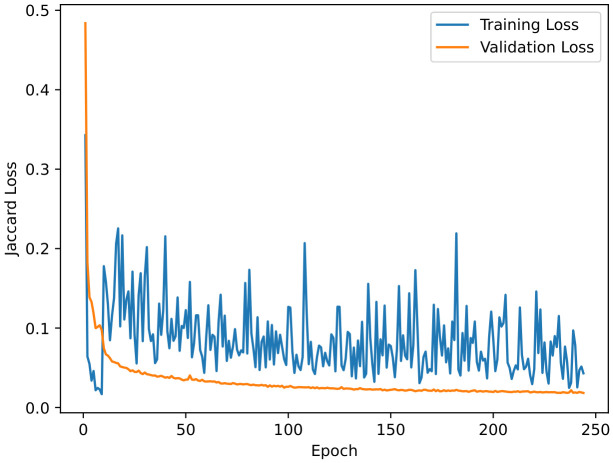
Training and validation loss.

The model is trained with a batch size of 64 over 243 epochs. Although we observed no overfitting of the model, as can be seen in the loss plot Figure A1 and still saw a decrease in validation loss, we stopped the training run after 243 epochs. We stopped the run because the validation loss curve showed, that no significant gains were to be expected if we had continued the training. Training was performed on an RTX 3090 (contactless) and RTX A6000 (pressed), with the main bottleneck being I/O speed.

##### Training Schedule

We implement a custom warm-up strategy for the initial 10 epochs, during which we train on the dataset without image augmentations. This phase utilizes patch sizes that are double the minimum and maximum values, as well as pixel offsets that are also twice the minimum and maximum values. This is inspired by curriculum learning, and helps the model to learn the foundational pattern, before increasing the difficulty to achieve the best performance also for minute artifacts. A more detailed analysis of the model’s training behavior is given in Appendix A.

##### Model Size

The model’s architecture consists of three main components: the encoder, the decoder, and the segmentation head. The following Table 1 provides a detailed breakdown of the parameters and FLOPs for each part of the model:

As seen in Table 1, the decoder dominates the compute cost, while the segmentation head has minimal impact.

### 2.3. Mosaicking Artifact Score

The segmentation mask alone is not directly usable for automated quality control over large datasets, since it provides spatial information rather than a scalar judgment. We therefore introduce a scalar mosaicking artifact score that summarizes the segmentation output into a single value, enabling automated decisions (e.g., flag for review, reject, accept) without manual inspection.

#### 2.3.1. Definition

The score *S* is defined as

(1) $$\begin{array}{cc} S & :=\underset{\mathrm{patch}\;\mathrm{contribution}}{\underset{︸}{(\sum_{i=1}^{n}\left(b_{patch}+w_{patch,i}\cdot h_{patch,i}\right)}}+\\ \; & \;c\cdot \underset{\mathrm{line}\;\mathrm{contribution}}{\underset{︸}{\left(\sum_{j=1}^{m}\left(s_{height}\cdot w_{line,j}\right)+\sum_{k=1}^{o}\left(s_{width}\cdot h_{line,k}\right))\right)}}\cdot \\ \; & \;\frac{100}{s_{width}\cdot s_{height}},\\ b_{patch} & :=b\cdot \frac{s_{width}\cdot s_{height}}{100}. \end{array}$$

The variables are defined in Table 2.

#### 2.3.2. Physical Interpretation

Each detected patch artifact incurs a fixed base cost in addition to a cost proportional to its area. This ensures that even small but unambiguous patch detections contribute a non-trivial amount to *S*. Because $b_{patch}$ is normalized by the mask area before the trailing $100/(s_{width}\cdot s_{height})$ factor, the base penalty contributes exactly *b* score units per detected patch, independent of image resolution.

Moreover, larger misaligned patches indicate more severe stitching failures and contribute proportionally more to the score. After the normalization factor, this term is expressed as a percentage of the image area.

And finally, vertical and horizontal line artifacts are penalized by their length along the corresponding image dimension, scaled by the weighting factor *c*. The product $s_{height}\cdot w_{line,j}$ (and analogously for horizontal lines) approximates the bounding-box area a line artifact would occupy if extended fully across the image. The factor *c* then attenuates this contribution to reflect that single-pixel-wide line artifacts perceptually disturb a much smaller region of the fingerprint than their bounding-box would suggest.

The factor $100/(s_{width}\cdot s_{height})$ at the end of the equation makes the score resolution-independent. Any pixel-counted contribution is expressed relative to the total image area in percent. As a consequence, the score of a 224×224 patch detection is directly comparable to that of a higher-resolution input.

#### 2.3.3. Parameter Choices for *b* and *c*

The constants b=5 and c=0.025 were chosen by design. b=5 was selected so that a single detected patch alone always produces S≥5, regardless of the patch’s pixel area. This gives a natural, interpretable detection threshold of one full patch detection. The value 5 (rather than, e.g., 1) provides headroom so that small spurious line activations do not by themselves cross the patch-equivalent threshold.

c=0.025 was chosen such that a line artifact spanning the entire image (i.e., $w_{line}=s_{width}$ or $h_{line}=s_{height}$) contributes $c\cdot s_{width}\cdot s_{height}\cdot 100/\left(s_{width}\cdot s_{height}\right)=2.5$ score units. This means a full-image line artifact contributes half as much as a single patch, reflecting that line artifacts typically affect a much thinner region than patch displacements.

These values express the design preference that patch displacements are more severe than line shifts of comparable extent. Their absolute values can be adapted to the operational needs of a specific pipeline. Calibrating *b* and *c* from a curated set of annotated real artifacts is left to future work and is discussed as a limitation in Section 4.6.

## 3. Experiments and Results

### 3.1. Model Performance

We evaluated the performance of both the contactless-trained (CL) and pressed-trained (PR) models in detecting fingerprint mosaicking artifacts using standard segmentation metrics: Intersection over Union (IoU), F1 Score, F2 Score, Accuracy, Recall, and the mean difference between predicted and ground-truth mosaicking artifact score. Evaluation was conducted on multiple datasets, including the Weissenfeld contactless dataset, NIST 300a slap and rolled fingerprints, and the PRD-1-Test, PRD-2, and ROD-1 datasets for pressed and rolled inputs.

Note that segmentation masks for mosaicking artifacts are highly imbalanced. Even strongly affected images have artifact pixels covering only a small fraction of the total mask area. As a result, pixel-wise accuracy is dominated by the (correctly classified) background and saturates near 1.000. For this reason, we treat accuracy as a sanity check only and rely primarily on IoU, F1, and F2 (which weight recall more heavily) for assessing detection quality.

Table 3 details the performance of the contactless-trained (CL) and pressed-trained (PR) models. Both models achieve excellent results in their native modality. The CL model scores an IoU of 0.982 on contactless data, while the PR model achieves an IoU of 0.977 on pressed data, with low mean artifact score differences of 0.264 and 0.355, respectively.

The models also show useful cross-modality generalization. The CL model transfers effectively to slap prints (NIST 300a), maintaining a high IoU of 0.959. As expected, generalization to heavily distorted rolled fingerprints is more challenging: the CL model’s IoU on rolled prints drops to 0.908, and the PR model’s to 0.931. Even in these more difficult cases, however, the mean score differences (1.061 for CL and 0.815 for PR) remain well below the patch-equivalent threshold of 5 (defined by patch weight *b*), indicating that the average per-image score error stays within a sub-detection regime across the modalities tested.

**Figure 5:**
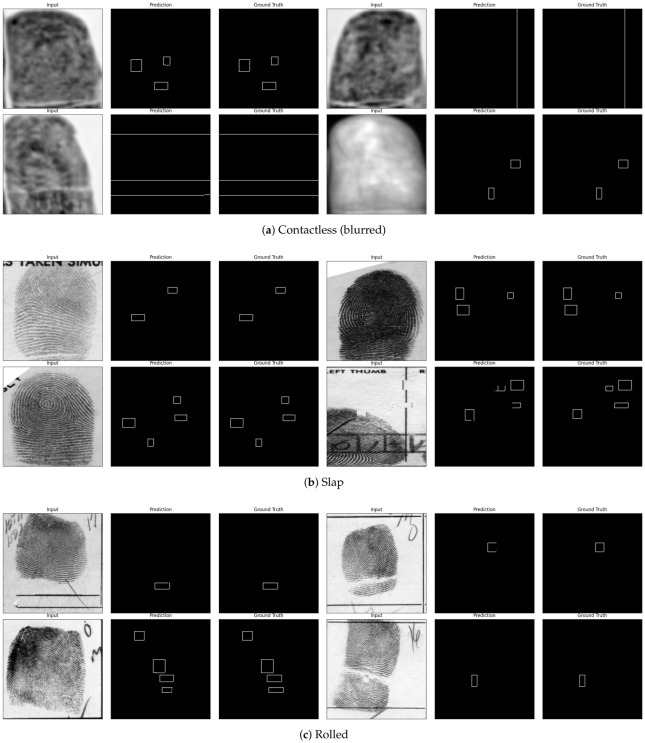
Example predictions of the CL model. Each row is a triplet showing, from left to right, the input image, the model’s predicted segmentation mask, and the synthetic ground-truth mask. (**a**) contactless prints (input images are blurred for privacy); (**b**) slap prints (NIST 300a); (**c**) rolled prints (NIST 300a).

Figure 5 shows example predictions of the CL model on contactless, slap, and rolled data. Each row is a triplet of input image (left), model output (middle), and ground truth (right).

**Figure 6:**
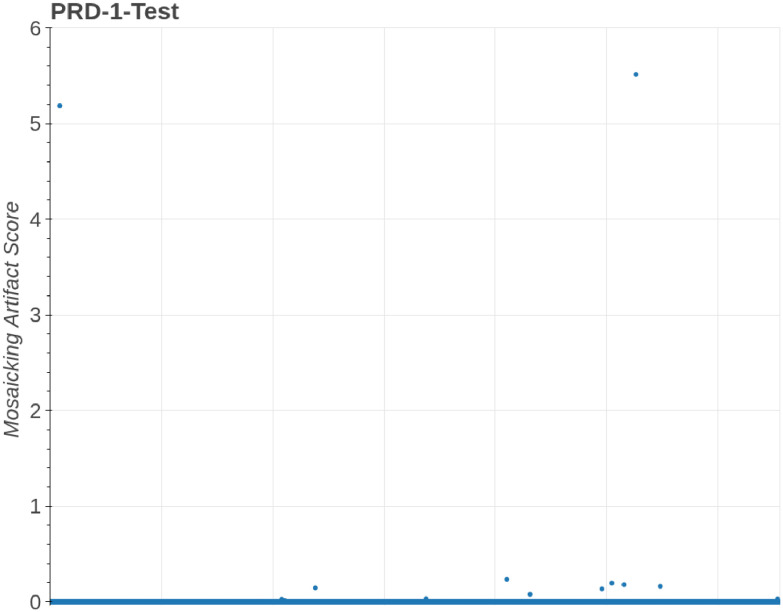
Distribution of the mosaicking artifact score on the PRD-1-Test dataset. Most images score near zero; the few elevated scores cluster around the patch weight b=5 and around 2b, reflecting the patch-count banding discussed in Section 3.1.

The distribution of the mosaicking artifact score for PRD-1-Test is shown in Figure 6. The corresponding distributions for ROD-1 and for a third dataset captured with a thin-film transistor (TFT) sensor are provided in Figure A5. The scores tend to cluster into at most three bands: most samples near zero, a small set around the patch-equivalent threshold of 5, and a few outliers near twice that value. For PRD-1-Test, only two samples fell into the second band and none into the third, corresponding to a false-positive rate of 0.061% at the patch-equivalent threshold.

**Figure 7:**
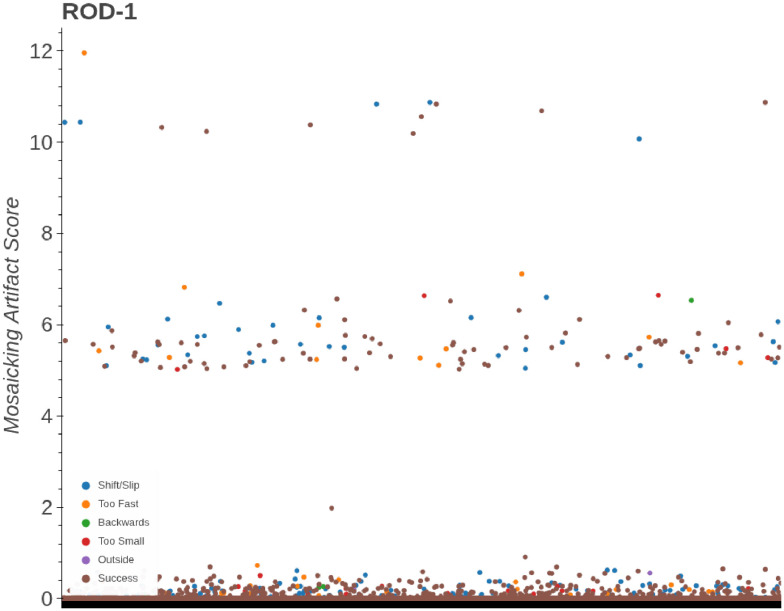
Distribution of the mosaicking artifact score on the ROD-1 dataset, with points colored by the operator-assigned sensor error code (see the list in Section 3.1). The same patch-count banding as in Figure 6 is visible. The error codes do not map one-to-one to mosaicking artifacts; both Shift/Slip and Success codes appear at the highest band.

The distribution of the mosaicking artifact score for ROD-1 colored with sensor error codes is shown in Figure 7. The observed errors are:

- Finger has been shifted or slipped during rolling. (Blue)
- Finger has been rolled too fast or an image has been missed. (Orange)
- Finger has been rolled backwards contrary to the determined roll direction. (Green)
- Size of rolled fingerprint is too small. (Red)
- Fingerprint was rolled outside the roll capture area. (Purple)
- Fingerprint was recorded without errors. (Brown)

Here, the separation into the three bands is more pronounced. The sensor error code does not map directly to a mosaicking artifact: a non-trivial fraction of samples in the highest band carries the Success code, and Shift/Slip is also frequent in both the highest and the second band. Figure A6 additionally analyzes the ROD-1 dataset by finger, providing further insights into per-identity behavior.

### 3.2. Baseline Comparison

To our knowledge, no publicly available model is dedicated to detecting fingerprint mosaicking artifacts, which precludes a direct head-to-head comparison with prior work on the exact same task. Therefore, we ran a controlled comparison against five segmentation baselines that vary either the decoder or the encoder while keeping the rest of the pipeline fixed. The set includes a plain UNet decoder paired with the same ResNeSt-50d encoder, three further widely used decoder families on the same encoder (MAnet [32], Linknet [33], FPN [34]), and one encoder swap (UNet on top of a standard ResNet-50 [35]).

All six configurations were trained under identical conditions: the NIST SD302d plain/press subset [36], 30 epochs, batch size 16, image resolution 224×224, learning rate $1.7\times 10^{-3}$, SGD with momentum 0.9, a combined BCE + 0.5· Jaccard loss, and two warm-up epochs (as in the main training schedule, but proportionally shorter). The same synthetic-artifact pipeline (Section 2.2.3) was used to generate the training signal. We emphasize that this short training schedule is chosen to make the comparison feasible across six configurations, not to reproduce the main results of Section 3.1; the absolute metrics in Table 4 are therefore lower than those in Table 3, and the table should be read for the relative ordering of architectures.

Three observations follow from Table 4. First, the proposed UNet++/ResNeSt-50d combination is best on every reported metric. Against the closest baseline (a plain UNet on the same encoder), the proposed model improves IoU by +0.049 (+6.6% relative), F2 by +0.048 (+5.8%), and recall by +0.058 (+7.2%); the mean score difference is also slightly lower. The nested skip pathways of UNet++ therefore provide a measurable, if modest, gain over a plain UNet on this task. Second, the remaining decoders trained on the same encoder (MAnet, Linknet, FPN) lag substantially under the same budget. We do not interpret this as a categorical claim that these architectures are unsuited to the task—with longer training and family-specific tuning, some of the gap may close—but it does show that the UNet/UNet++ family is the most data-efficient choice in this setting. Third, swapping the encoder from ResNeSt-50d to a plain ResNet-50 while keeping the UNet decoder fixed causes a much larger collapse in performance (IoU drops from 0.742 to 0.273) than swapping the decoder while keeping the encoder fixed. The encoder choice therefore appears to matter more than the decoder choice for detecting locally coherent disturbances against a textured ridge background, which is consistent with the architectural rationale given in Section 2.2.4 for using a split-attention encoder.

We note two caveats. The baselines are run for 30 epochs rather than the 243 epochs of the main model, so the absolute IoU/F2 values are lower than in Table 3 for every configuration, including the proposed one. The relative ordering between architectures is the substantive output here. The loss function used for the baseline runs is also slightly different from the main run (BCE + 0.5· Jaccard rather than pure Jaccard), to give plain UNet a fair chance with a well-tuned, stable loss; this also affects the absolute MSD values.

### 3.3. Model Robustness

We conducted a robustness analysis to test generalization across acquisition conditions and to evaluate the impact of non-mosaicking visual artifacts. A second model (PR) was trained with identical architecture but different hyperparameters on 26,240 FTIR-based pressed fingerprint images, substantially fewer than the 245,193 contactless images used for CL.

**Figure A2:**
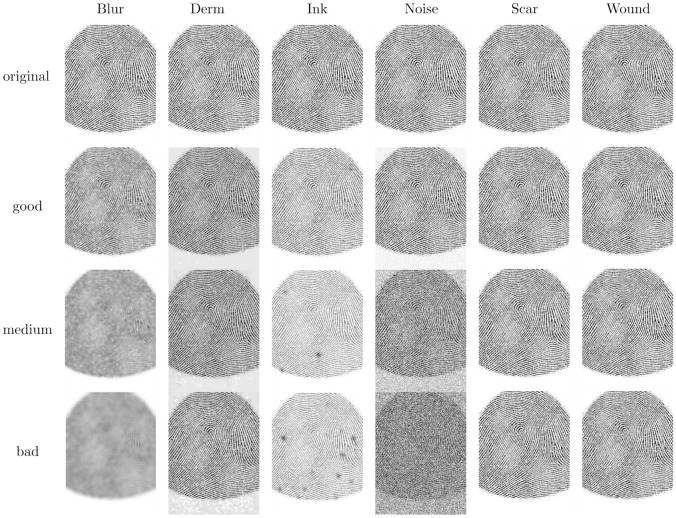
Visualizations of different fingerprint alterations.

**Figure A5:**
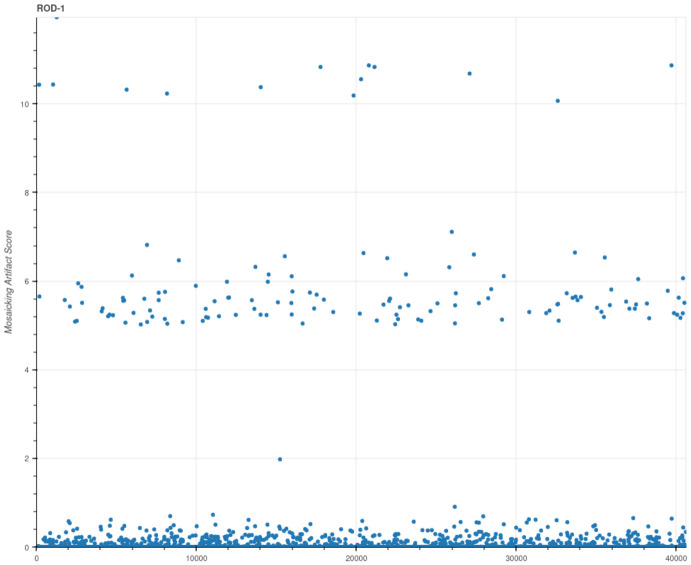
Mosaicking artifact score distribution of PR model for ROD-1.

To evaluate sensitivity to non-mosaicking distortions, both models were tested on 100 synthetically altered fingerprints from SFinGe using SynColFinGe. Alterations included different intensities of noise, wounds, scars, ink variations, and skin problems. Figure 1 shows sample images with medium ink variation, light scarring, and extensive noise. The full set of alteration types is shown in Figure A2.

Table 5 reports the maximum, median, mean, and standard deviation of the mosaicking artifact score across all alteration types and intensity levels. In nearly all cases, both models produce scores well below the patch-equivalent threshold of 5. The only exceptions are a single CL-model result and three PR-model results in the medium-noise condition, which slightly exceed the threshold.

Under these synthetic non-mosaicking distortions, both models therefore appear robust to typical image-quality alterations, with only rare false activations on a small subset of medium-noise images. We note that this evaluation is itself based on synthetic alterations (SynColFinGe); behavior on the corresponding real distortions is expected to follow the same trend but has not been directly verified here.

### 3.4. Effect of Mosaicking Errors on Equal-Error Rate

To assess how mosaicking errors affect fingerprint recognition accuracy, we measured the Equal-Error Rate (EER) using three commonly used Automated Biometric Identification Systems (ABIS):

1. FingerNet + SourceAFIS: Combines open-source, deep learning-based segmentation and minutiae extraction (FingerNet [37]) with a fast and accurate open-source matcher (SourceAFIS [38]).
2. NBIS: A widely used NIST toolset [39] including MindTCT for minutiae extraction and Bozorth3 for matching.
3. Innovatrics’ IDKit: A commercial solution known for high recognition accuracy [40].

The EER analysis was performed on a subset of 500 contactless fingerprint images drawn from 50 distinct fingers (10 images per finger). This yields 4500 genuine comparisons (50·102) and 50·49·10·10/2=122,500 impostor comparisons per condition. For each ABIS and each condition (none/small offset/large offset), all pairwise comparisons were computed, and the EER was determined as the operating point at which the false match rate equals the false non-match rate. The reported EER values should be interpreted with the corresponding statistical uncertainty. For a binomial proportion estimator with ∼4500 genuine comparisons, the half-width of an exact 95% confidence interval at the observed rates is on the order of ±0.2–0.3 percentage points for the SourceAFIS and IDKit pipelines, and on the order of ±0.6 percentage points for the Bozorth3 pipeline.

We categorized mosaicking errors into two groups based on the offset ratio of the artifacts relative to the image dimensions:

1. Small Offsets (1–2%): Minor displacements. For a 1.5×2.5 cm fingerprint, this translates to about 2–5 pixels horizontally (0.15–0.3 mm) and 4–9 pixels vertically (0.25–0.5 mm).
2. Large Offsets (2–7%): More severe displacements. For the same fingerprint, this implies 5–20 horizontal pixels (0.5–1.05 mm) and 9–34 vertical pixels (0.5–1.75 mm).

We evaluated each ABIS by computing the EER across three conditions: original (unaltered) fingerprints, fingerprints with small mosaicking offsets, and fingerprints with large mosaicking offsets.

Results, shown in Table 6, indicate that even small mosaicking artifacts increase the EER noticeably. Larger artifacts further degrade performance across all systems. The commercial solution (IDKit) was the most robust, followed by the NBIS tools, while the FingerNet+SourceAFIS pipeline showed the highest sensitivity to these errors. The relative EER increase from the unaltered to the small-offset condition is more than 2× for the SourceAFIS pipeline, ∼1.4× for Bozorth3, and ∼2.3× for IDKit—changes that exceed the statistical uncertainty in all three cases.

These results show that even low-level mosaicking artifacts can impair fingerprint matching.

## 4. Discussion

### 4.1. Model Performance

Each model performs best on the modality it was trained on: the CL model on contactless data and the PR model on pressed data. The CL model also transfers well to slap images, which is a different recording modality, indicating useful generalization to out-of-distribution data. This is notable given the difficulty of the NIST 300a dataset, where scanned ink prints are often overlaid with text or other obstructions. Both models also retain high performance on rolled ink prints (NIST 300a rolled) and on live-scanned TFT data (ROD-1), with only minor drops in IoU.

As shown in Table 3, the classical segmentation metrics (IoU, recall, F1, F2) co-vary with the mean absolute difference between the predicted and ground-truth mosaicking artifact score. For both models, this score difference remains well below the patch-equivalent threshold (b=5) from Equation (1).

The score distributions on PRD-1-Test, ROD-1, and the supplementary TFT dataset also show that false positives are rare. On PRD-1-Test, the false-positive rate at the patch-equivalent threshold is only 0.061%, suggesting that the framework can be applied at scale without flagging an unmanageable number of artifact-free images. We note that this rate is conditioned on the PRD-1-Test distribution, which is assumed to be (and visually appears) artifact-free; an analogous rate on annotated real data is left as future work.

**Figure A6:**
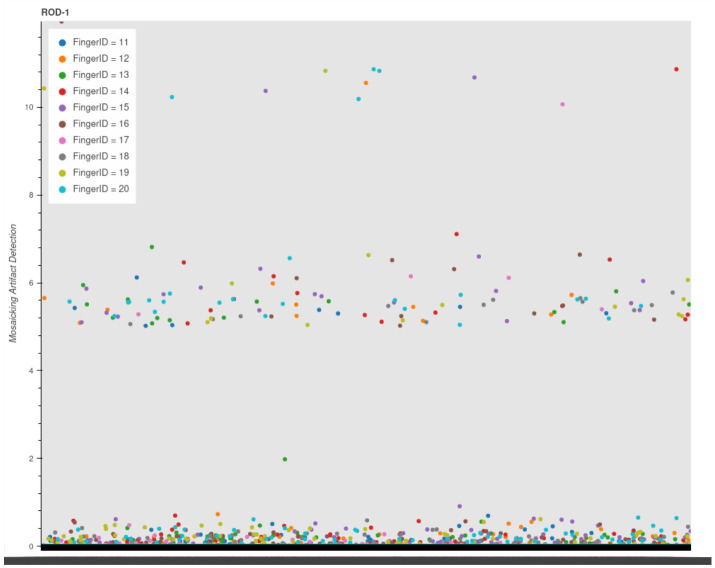
Mosaicking artifact score distribution of PR model for ROD-1, with finger position (FGP) as color label.

The predicted scores naturally cluster into a small number of bands corresponding to multiples of the patch weight, as visible in Figure 6, Figure 7 and Figure A6. This banding suggests a coarse, interpretable ranking of artifact severity: zero patches, one patch, two patches, and so on.

### 4.2. Model Robustness

Table 5 shows that both models behave consistently across the tested fingerprint alterations, with artifact scores almost always well below the patch-equivalent threshold of 5. The models therefore do not appear to confuse skin defects, ink variations, noise, scars, or wounds with mosaicking artifacts.

The exceptions occur under medium noise. The CL model peaks at 5.18, marginally above the threshold, on a single image out of 100. The PR model peaks at 10.41 on three images out of 100. These rare outliers leave the means, medians, and standard deviations essentially unchanged.

Across the tested conditions, both models behave stably; the PR model is slightly more consistent under high noise. We emphasize that this robustness evaluation uses synthetic alterations (SynColFinGe). Real-world distortions are expected to behave similarly, but a direct evaluation on annotated real distortions remains future work.

### 4.3. Impact on EER and Dataset Acquisition

As shown in Table 6, the introduction of mosaicking artifacts increases the Equal Error Rate (EER) across all three tested ABIS pipelines. Even small artifacts can roughly double the EER of the most sensitive pipeline, with relative increases that exceed the statistical uncertainty of the experiment (Section 3.4). Integrating an artifact detector during acquisition would therefore be expected to improve image quality and downstream ABIS performance.

This underlines the value of careful data acquisition, especially in high-security contexts. Modern sensors have reduced artifact frequency but not eliminated it. Embedding a dedicated detector in the acquisition pipeline could act as a complementary safeguard, similar in function to NFIQ-style quality checks but specifically targeting mosaicking artifacts.

### 4.4. Mosaicking Artifact Score

The proposed mosaicking artifact score provides a compact way to quantify the severity of fingerprint mosaicking artifacts. The weights b=5 for patch artifacts and c=0.025 for line artifacts express the relative impact of these two failure modes by design (Section 2.3.3). The EER experiments in Section 4.3 provide indirect, downstream evidence that artifacts at this scale matter for biometric matching; they do not, however, constitute an empirical calibration of *b* and *c* against expert severity labels, which we list as a limitation in Section 4.6.

For cases where a patch-based misalignment is plausible, we propose linking the detection threshold to the patch weight *b* of Equation (1): an image is flagged once the model finds at least one closed patch.

As noted in Section 4.1, the mean score difference in Table 3 closely tracks the classical segmentation metrics (IoU, recall, F1, F2). The score therefore serves a dual role: it summarizes model quality on a validation set into a single number, and it provides an automated, per-image severity estimate at inference time.

An additional analysis of a thin-film transistors (TFT) sensor based dataset can be found in Appendix C.

### 4.5. Research Impact

A reliable detector for mosaicking artifacts could be useful in real-world biometric pipelines. Agencies in national security, law enforcement, and border control could potentially improve identification accuracy by integrating such a detector into their acquisition and verification stages. Automatically flagging artifact-affected images would reduce the risk of misidentification and improve database hygiene, though the magnitude of this improvement in a given operational pipeline would still need to be measured against that pipeline’s specific artifact statistics.

The proposed score could also be considered as a quality-control measure across different fingerprint acquisition devices. Its observed behavior across modalities makes it a candidate for broader use, and could help inform sensor and stitching-algorithm development.

Current quality standards, such as NFIQ 2 are designed for pressed fingerprints and are often applied to rolled fingerprints, which present different challenges. Rolled fingerprints are prone to deformation and to mosaicking artifacts that arise from the interplay of hardware and stitching algorithms. Such artifacts can create or shift minutiae and lead to identification errors, which is particularly problematic in central fingerprint databases. Automated tools that flag these artifacts during acquisition and processing can reduce reliance on manual inspection in applications such as asylum screening and identity verification.

### 4.6. Limitations

We close the discussion with a summary of the main limitations of this study. Each limitation also defines the corresponding direction for follow-up work.

The model is trained and quantitatively evaluated on artificially generated patch and line artifacts. Section 2.2.3 provides a qualitative comparison between our synthetic artifacts and a real failure observed in our acquisition setup, and Section 4.1 reports indirect support: high-score outliers on ROD-1 concentrate on images that operators flagged as shift/slip or as “success after rolling”. We do not, however, have a sufficiently large pool of pixel-level annotated real mosaicking artifacts to evaluate the model against a real-artifact ground truth in a fully quantitative manner. Constructing such a benchmark would require multi-sensor, multi-operator annotation under expert supervision, which is itself a substantial undertaking. Statements about operational performance in this paper should therefore be read as conditional on this synthetic-only validation.

The values b=5 and c=0.025 in Equation (1) are chosen by design (Section 2.3.3) and are not the result of an empirical calibration against expert annotations or operational severity labels. Once real-artifact data with per-image severity labels is available, *b*, *c*, and the threshold can be re-fitted to maximize agreement with the labels. Data-driven or learning-based scoring approaches that replace the closed-form score with a regression model trained on annotated severity labels are a natural next step.

We adopt ResNeSt-50d as the encoder and UNet++ as the decoder without architectural modification. This is a deliberate methodological choice (Section 2.2.4). The contribution of this work lies in the self-supervised training pipeline, the synthetic-artifact generation strategy, the mosaicking artifact score, and the cross-modality evaluation, not in the network architecture itself. The baseline comparison in Section 3.2 shows that this combination outperforms a plain UNet, three further decoder families, and a non-split-attention encoder under matched training conditions, but the improvement over the closest baseline (plain UNet with the same encoder) is modest. A full search over architectures, encoders, and self-supervised training schedules is left as future work.

### 4.7. Outlook

A promising direction for future work is the supervised fine-tuning of our self-supervised model on real, annotated mosaicking artifacts. The self-supervised pipeline already provides an effective initialization; fine-tuning on real artifacts would close the remaining gap between simulated and real failure modes. This depends on the construction of the annotated benchmark discussed above.

A second direction is the integration of the detector into edge devices or directly into fingerprint sensors. Running the detector at the point of acquisition would allow errors to be caught in real time, improving the quality of the data before it enters the downstream processing pipeline.

Discussions on updating the NFIQ 2 standard [41] to formally cover contactless and rolled fingerprints have recently begun. Our framework could be a useful component in that effort, especially for the detection and correction of mosaicking and related artifacts.

Finally, future contactless acquisition systems may capture multiple images from different angles to achieve interoperability with rolled fingerprints. Mosaicking will play a central role in such systems, which makes reliable artifact detection a building block for the next generation of fingerprint acquisition.

## 5. Conclusions

We have presented a deep learning-based framework for detecting hard mosaicking artifacts in fingerprint images. The framework uses a self-supervised learning paradigm, which makes it possible to train on large unlabeled fingerprint datasets without manual artifact annotation. To complement the segmentation output, we introduced a mosaicking artifact score that provides a scalar measure of artifact severity and enables automated evaluation and prioritization of fingerprint images for further processing or rejection. The score has an explicit physical interpretation; its parameters *b* and *c* are justified by design rather than calibrated against expert annotations (Section 2.3.3).

On synthetic mosaicking artifacts, the proposed framework performs consistently across contactless, rolled, and pressed fingerprints and across multiple data sources. A controlled baseline comparison (Section 3.2) places this performance in the context of five common segmentation configurations and indicates that the encoder choice matters more than the decoder choice on this task. A fully quantitative validation against a curated set of manually annotated real artifacts remains an open follow-up (Section 4.6).

Within these limits, the framework is a useful step toward more reliable fingerprint-based biometric systems. By flagging and excluding mosaicking-affected images, it can contribute to more accurate fingerprint matching and verification, and ultimately to more secure biometric authentication pipelines.

## Figures and Tables

**Table 1:** Model Component Breakdown: Parameter Count and Floating-Point Operations (FLOPs). Values are expressed in millions (M) and billions (G).

Part	Parameter	FLOPs
Encoder	25.4 M	21.7 G
Decoder	25.5 M	159.8 G
Segmentation Head	145	28.9 M
Total	50.9 M	181.6 G

**Table 2:** Definitions of the variables used in the mosaicking artifact score (Equation (1)).

Symbol	Meaning
$s_{width},s_{height}$	Width and height of the segmentation mask in pixels.
*n*	Number of detected patch-shaped (2D, closed) artifact regions.
*m*	Number of detected vertical line artifacts.
*o*	Number of detected horizontal line artifacts.
$w_{patch,i},h_{patch,i}$	Width and height (in pixels) of the *i*-th detected patch.
$w_{line,j}$	Width (in pixels) of the *j*-th vertical line artifact.
$h_{line,k}$	Height (in pixels) of the *k*-th horizontal line artifact.
*b*	Patch base weight (dimensionless), see Section 2.3.3.
$b_{patch}$	Per-patch base contribution, scaled to mask area.
*c*	Line-artifact weighting factor, see Section 2.3.3.

**Table 3:** Model performance of the contactless-trained model (CL) and the pressed-trained model (PR) measured via intersection-over-union (IoU), F1 and F2 score, pixel-wise accuracy, recall, and mean mosaicking artifact score difference (Mean Score Dif.) for contactless data (first row), contact-based rolled data (NIST 300a rolled), contact-based slap data (NIST 300a slap), pressed data from the test set (PRD-1-Test), pressed data acquired after the training cutoff (PRD-2), and rolled data (ROD-1). Accuracy saturates near 1.000 due to severe class imbalance in the segmentation masks; IoU, F1, and F2 are the more informative quality indicators.

	Dataset	IoU	F1	F2	Accuracy	Recall	Mean Score Dif.
CL	Weissenfeld et al. [21]	0.982	0.991	0.990	1.000	0.989	0.264
	NIST 300a slap	0.959	0.979	0.975	1.000	0.972	0.483
	NIST 300a rolled	0.908	0.952	0.940	1.000	0.932	1.061
PR	PRD-1-Test	0.977	0.988	0.987	1.000	0.986	0.355
	PRD-2	0.978	0.989	0.988	1.000	0.987	0.351
	ROD-1	0.931	0.964	0.957	1.000	0.952	0.815

**Table 4:** Baseline comparison on NIST SD302d (plain/press subset) under matched training conditions (30 epochs, batch 16, image 224×224, LR $1.7\times 10^{-3}$, SGD with momentum 0.9, BCE + 0.5· Jaccard loss). MSD denotes the mean mosaicking artifact score difference. The reduced training budget makes the absolute numbers not directly comparable to Table 3; the table is intended to compare architectures, not to re-evaluate the main result. Bold incidates best performing entry.

Configuration	IoU	F1	F2	Accuracy	Recall	MSD
UNet++/ResNeSt-50d (proposed)	**0.791**	**0.883**	**0.873**	0.999	**0.866**	**3.70**
UNet/ResNeSt-50d	0.742	0.852	0.825	0.999	0.808	3.78
MAnet/ResNeSt-50d	0.617	0.763	0.767	0.998	0.769	4.26
Linknet/ResNeSt-50d	0.376	0.547	0.462	0.997	0.418	8.26
FPN/ResNeSt-50d	0.273	0.429	0.532	0.992	0.634	8.21
UNet/ResNet-50	0.273	0.429	0.368	0.996	0.336	8.43

**Table 5:** Robustness ablation results of the model trained on contactless images (CL Model) and the model trained on contact-based images (PR Model). Columns indicate no modification (No), skin damage (Skin), ink problems (Ink), added image noise (Noise), added scars (Scar), and added wounds (Wounds). The arrows indicate low (↓), medium (_~_), and high (↑) intensity of the image modification. The table presents the maximum value (max), median value, mean value, and standard deviation (std) of the mosaicking artifact score.

		No	Skin		Ink			Noise			Scar			Wounds		
			↓	_~_	↑	_~_	↑	↓	_~_	↑	↓	_~_	↑	↓	_~_	↑
CL Model	max	1.12	1.13	0.81	1.19	1.09	1.07	0.44	5.18	0.56	0.93	1.14	1.31	1.11	1.08	1.35
	median	0.01	0.03	0.01	0.02	0.03	0.00	0.00	0.00	0.00	0.02	0.04	0.02	0.02	0.03	0.08
	mean	0.15	0.15	0.10	0.14	0.15	0.11	0.03	0.09	0.05	0.15	0.15	0.14	0.14	0.18	0.23
	std	0.26	0.25	0.18	0.24	0.25	0.24	0.08	0.56	0.12	0.26	0.24	0.26	0.24	0.26	0.32
PR Model	max	0.00	0.00	0.00	0.00	0.00	0.01	0.00	10.41	0.13	0.00	0.00	0.00	0.00	0.00	0.21
	median	0.00	0.00	0.00	0.00	0.00	0.00	0.00	0.00	0.00	0.00	0.00	0.00	0.00	0.00	0.00
	mean	0.00	0.00	0.00	0.00	0.00	0.00	0.00	0.24	0.00	0.00	0.00	0.00	0.00	0.00	0.00
	std	0.00	0.00	0.00	0.00	0.00	0.00	0.00	1.27	0.01	0.00	0.00	0.00	0.00	0.00	0.03

**Table 6:** Equal-Error Rate (EER) for three ABIS pipelines on the 500-image/50-finger contactless subset (4500 genuine, 122,500 impostor comparisons per condition), reported for original images and for images with small and large mosaicking offsets.

Artifacts	SourceAFIS [%]	Bozorth3 [%]	IDKit [%]
None	0.43	3.97	0.38
Small Offset	0.91	5.41	0.88
Large Offset	0.99	4.82	0.88

**Figure A3:**
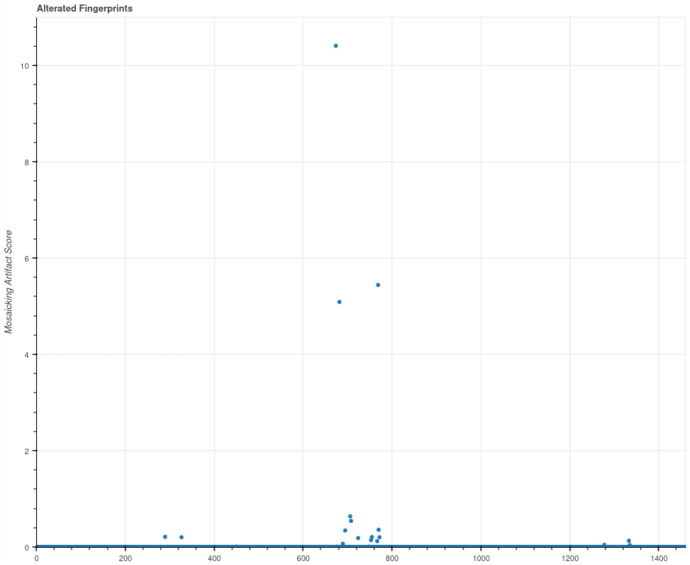
Mosaicking artifact score of PR model on altered fingerprint.

**Figure A4:**
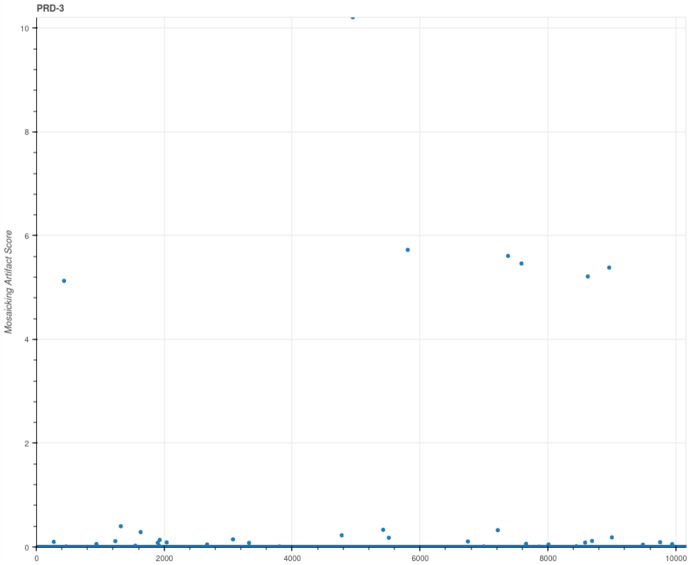
Mosaicking artifact score distribution of PR model for PRD-3 dataset.

## Data Availability

Data is unavailable due to privacy restrictions.

## Appendix A. Loss Behavior

The learning curves presented in Figure A1 depict the model’s performance, measured by Jaccard loss, on both the training and validation sets over 243 epochs. Initially, both curves exhibit a rapid descent, indicating effective learning in the early stages of training. The training loss continues to decrease, albeit at a slower rate and with oscillations, throughout the entire training duration, eventually reaching a low value. The large oszillations of the training loss indicate the increased challenges connected with strong data augmentation. Furthermore, the fast and large decrease in the training loss suggests the model is achieving a good fit to the training data. However, the validation loss plateaus significantly earlier, approximately between epochs 10 and 20, remaining relatively flat thereafter with minor fluctuations. This divergence between training and validation loss signifies the onset of reduced gains from further training. The training was stopped at 243 epochs as further training is assumed to yield only diminishing returns in validation performance, while simultaneously increasing the risk of overfitting. The minimal reduction in validation loss observed after the initial plateau indicates that the model had largely exhausted its capacity to generalize to unseen data within the given hyperparameter configuration and training data provided.

## Appendix B. Synthetic Finger Alterations

In Figure A2, a depiction of every available fingerprint alteration technique used in this work is shown. These techniques are categorized into simulations of skin-specific conditions, external artifacts, and image quality degradations.

### Appendix B.1. Skin-Specific Alterations

- Derm (Dermatological Issues): This category simulates the presence of skin conditions that can affect the clarity and visibility of fingerprint ridges. The “Derm” alteration introduces localized blurring and reduces contrast in affected areas, mimicking the impact of conditions such as eczema or dryness on fingerprint patterns.
- Scar: This alteration simulates the presence of scars, which disrupt the natural flow of fingerprint ridges. Scars are modeled as linear or irregularly shaped regions with altered ridge orientations and reduced contrast, representing the tissue damage and subsequent healing process.
- Wound: Open wounds are simulated by introducing areas of complete ridge disruption, represented as localized regions of high contrast and irregular texture. The “Wound” alteration signifies areas where the fingerprint pattern is temporarily or permanently obscured due to injury.

### Appendix B.2. External Artifacts

Ink: This alteration simulates the presence of ink stains on the fingertip, which can occur in scenarios involving traditional fingerprinting methods or accidental ink exposure. Ink stains are modeled as localized areas of increased darkness and obscured ridge details, reflecting the opaqueness and potential smudging of ink.

### Appendix B.3. Image Quality Degradations

- Blur: This set of alterations simulates various levels of blurriness, from slight softening to significant defocus. The “Blur” technique degrades the image by applying Gaussian blur to the fingerprint, simulating the effects of poor focus, motion, or low-quality capturing devices.
- Noise: This alteration simulates the presence of sensor noise, a common artifact in digital imaging. Noise is introduced as random variations in pixel intensity across the fingerprint image, mimicking the graininess or speckling that can occur due to limitations in sensor technology or low-light conditions.

These alterations allow for the generation of synthetic fingerprint images that exhibit a wide range of characteristics and imperfections observed in real-world contactless fingerprint data. The specific parameters of each alteration can be adjusted to control the severity and prevalence of the simulated effects, providing flexibility in generating datasets for various research and evaluation purposes. The following categories, “good”, “medium”, and “bad” are used to describe the severity.

### Appendix B.4. Mosaicking Artifact Score on Alterated Images

To further assess the robustness of our proposed model against common fingerprint alterations, we evaluated the Mosaicking Artifact Score on synthetically altered images. These alterations were applied to a set of 100 synthetically generated fingerprints from the SFinGe dataset. The distribution of the resulting Mosaicking Artifact Scores is visualized in Figure A3.

The scatter plot in Figure A3 displays the Mosaicking Artifact Score for each altered image. The *x*-axis represents the sample number, while the *y*-axis represents the calculated Mosaicking Artifact Score.

A significant concentration of data points is clustered around the zero mark on the *y*-axis. This indicates that for the majority of altered images, the model correctly did not detect any significant mosaicking artifacts, demonstrating its robustness to these alterations. There are a few notable outliers with higher Mosaicking Artifact Scores. Specifically, there are several points located between 5 and 6 and one outlier point significantly above 10. These outliers likely correspond to images where the alterations were either severe enough to mimic the appearance of mosaicking artifacts or where the model misidentified certain alterations as artifacts. Given that the threshold for detecting a mosaicking artifact is linked to the patch weight b, which is set to 5, these outliers above 5 could be considered false positives. The distribution of the scores suggests that the model is generally robust to the applied alterations, with only a small fraction of images producing scores that could potentially be considered false positives.

## Appendix C. Mosaicking Artifact Score Distribution

In addition to the PRD-1 and PRD-2 pressed fingerprint recordings datasets collected using a FTIR sensor, we have data collected from a thin-film transistors (TFT) based sensor. We call this dataset PRD-3, and it consists of 10,159 fingerprint recordings from 134 user. The users overlap with the users in PRD-1 and PRD-2.

The following Figure A4, Figure A5 and Figure A6 show the distribution of the mosaicking artifact error score for the TFT sensor and the ROD-1 dataset.

There, one can see that the score separates into three bands. One band with the majority of the samples sitting around zero, one band with only a few examples around the patch weight of 5 and then the final band with only a handful of outliers at twice the patch weight. Note also that the second and third band are less populated for the TFT-Pressed case in Figure A4 than for the rolled case in the following figures. This shows in the general lower mosaicking artifact scores.

The Appendix C also provide the distribution of the mosaicking artifact score for the ROD-1 dataset analyzed in terms of differences between fingers and also in terms of error types indicated during the acquisition process, such as slipping.

For the finger wise investigation of the mosaicking artifact score in Figure A6, there is no clear correlation between score and finger. Note, the naming scheme of the plot follows the NIST guidelines on the finger position (FGP), where 11 stands for the rolled right thumb print and 16 for the rolled left thumb print. The other numbers start with the index fingers (12 and 17) and end with the little fingers.
